# Functional characterization of a nanobody-based glycoprotein VI-specific platelet agonist

**DOI:** 10.1016/j.rpth.2024.102582

**Published:** 2024-10-03

**Authors:** Minka Zivkovic, Elisabeth Pols - van Veen, Vossa van der Vegte, Silvie A.E. Sebastian, Annick S. de Moor, Suzanne J.A. Korporaal, Roger E.G. Schutgens, Rolf T. Urbanus, Thrombocytopathy in the Netherlands (TiN) study group, Erik Beckers, Michiel Coppens, Jeroen Eikenboom, Louise Hooimeijer, Gerard Jansen, Roger Schutgens, Rolf Urbanus, Minka Zivkovic, SYMPHONY consortium, Emile van den Akker, Wala Al Arashi, Ryanne Arisz, Lieke Baas, Ruben Bierings, Maartje van den Biggelaar, Johan Boender, Anske van der Bom, Mettine Bos, Martijn Brands, Annelien Bredenoord, Laura Bukkems, Lex Burdorf, Jessica Del Castillo Alferez, Michael Cloesmeijer, Marjon Cnossen, Mariëtte Driessens, Jeroen Eikenboom, Karin Fijnvandraat, Kathelijn Fischer, Geertje Goedhart, Tine Goedhart, Samantha Gouw, Rieke van der Graaf, Masja de Haas, Lotte Haverman, Jan Hazelzet, Shannon van Hoorn, Elise Huisman, Nathalie Jansen, Alexander Janssen, Sean de Jong, Sjoerd Koopman, Marieke Kruip, Sebastiaan Laan, Frank Leebeek, Nikki van Leeuwen, Hester Lingsma, Moniek de Maat, Ron Mathôt, Felix van der Meer, Karina Meijer, Sander Meijer, Stephan Meijer, Iris van Moort, Caroline Mussert, Hans Kristian Ploos van Amstel, Suzanne Polinder, Diaz Prameyllawati, Simone Reitsma, Eliza Roest, Lorenzo Romano, Saskia Schols, Roger Schutgens, Rolf Urbanus, Carin Uyl, Jan Voorberg, Huan Zhang, Minka Zivkovic

**Affiliations:** 1 Center for Benign Haematology, Thrombosis and Haemostasis, Van Creveldkliniek, University Medical Center Utrecht, Utrecht University, Utrecht, the Netherlands; 2 Circulatory Health Research Center, University Medical Center Utrecht, Utrecht, the Netherlands; 3 Central Diagnostic Laboratory, University Medical Center Utrecht, Utrecht, the Netherlands

**Keywords:** diagnostic tests, glycoprotein, nanobodies, platelet activation, platelet function tests

## Abstract

**Background:**

Glycoprotein (GP)VI is a platelet-specific collagen receptor required for platelet activation during hemostasis. Platelet reactivity toward collagen is routinely assessed during diagnostic workup of platelet disorders. GPVI can be activated by inducing receptor clustering with suspensions of fibrillar collagen or synthetic cross-linked collagen-related peptide (CRP-XL). However, these suspensions are poorly standardized or difficult to produce. Nanobodies are small recombinant camelid-derived heavy-chain antibody variable regions. They are highly stable, specific, and ideal candidates for developing a stable GPVI agonist for diagnostic assays.

**Objectives:**

Develop a stable nanobody-based GPVI agonist.

**Methods:**

Nanobody D2 (NbD2) was produced as dimers and purified. Tetramers were generated via C-terminal fusion of dimers with click chemistry. Nanobody constructs were functionally characterized with light transmission aggregometry (LTA) in platelet-rich plasma and whole blood flow cytometry. Diagnostic performance was assessed in patients with inherited platelet function disorders with LTA and flow cytometry.

**Results:**

NbD2 was specific for human platelet GPVI. Dimers did not result in platelet activation in LTA or flow cytometry settings and fully inhibited CRP-XL-induced P-selectin expression and fibrinogen binding in whole blood and attenuated collagen-induced platelet aggregation in platelet-rich plasma. However, NbD2 tetramers caused full platelet aggregation, as well as P-selectin expression and fibrinogen binding. NbD2 tetramers were able to discriminate between inherited platelet function disorder patients and healthy controls based on fibrinogen binding, similar to CRP-XL.

**Conclusion:**

Nanobody tetramers to GPVI induce platelet activation and can be used to assess the GPVI pathway in diagnostic assays.

## Essentials

• The GPVI pathway is always assessed during platelet diagnostics.
• We developed a nanobody-based GPVI agonist to assess platelet function.
• Nanobody-based GPVI agonists performed similarly to CRP-XL.
• Nanobody tetramers can be used to specifically address the GPVI pathway.

## 1 Introduction

Glycoprotein (GP)VI is a megakaryocyte and platelet surface-specific membrane glycoprotein that is identified as the major activating receptor for collagen and also a receptor for other ligands, including fibrin [1]. Upon vessel wall injury, extravascular collagen is exposed to platelets in the circulation. Interaction of platelets with collagen through GPVI results in platelet activation and contributes to thrombus formation [2,3]. GPVI is a type I transmembrane receptor protein and belongs to the immunoglobulin (Ig)-like receptor family. It consists of 2 extracellular Ig domains (D1 and D2), a mucin-like stalk domain, and a short cytoplasmic tail containing calmodulin- and Src kinase-binding sites [2,3]. On the platelet surface, the signaling of GPVI depends on its association with the Fc receptor γ-chain, which contains an immunoreceptor tyrosine-based activation motif. The binding of collagen to GPVI induces receptor cross-linking, leading to the recruitment and activation of downstream signaling molecules, including the receptor tyrosine kinase SYK [4].

Activation of platelets through GPVI signaling is often assessed in diagnostic assays for platelet disorders. Different inherited and acquired disease-causing variants of GPVI are known, which include mutations in the *GP6* gene causing the bleeding disorder platelet-type 11, a mild to moderate bleeding disorder characterized by defective platelet activation and aggregation in response to collagen [[5], [6], [7]]. Also, platelets may be deficient in GPVI due to inherited or acquired loss of the protein, the latter through, eg, autoantibody-induced receptor shedding. For instance, the GPVI/Fc receptor γ-chain complex is absent on the platelet surface in anti–GPVI-associated immune thrombocytopenia [8]. Additionally, GPVI deficiency could be nonimmune and associated with Gray platelet syndrome [9]. Besides deficiency of GPVI, some patients have a normal surface expression of GPVI but present with a congenital or acquired GPVI-related signaling defect [10,11]. In these disorders, reduced collagen-induced platelet activation in diagnostic assays is a prominent feature. Secondary deficits in the response to collagen are observed in storage pool deficiencies (SPDs) [12], and since activation of the fibrinogen receptor after collagen stimulation depends on secondary activation through adenosine diphosphate (ADP) secretion or thromboxane A2 production, platelets can show a decreased response to collagen when thromboxane A2 or ADP secretion are decreased or absent [13].

Current agonists that are used in diagnostic assays for platelet function disorders, like aggregation assays and flow cytometry assays [14], include insoluble collagen fiber suspensions, the soluble agonist cross-linked collagen-related peptide (CRP-XL), and the snake venom tetramer protein convulxin. Only the latter 2 are specific to GPVI. Limitations of the GPVI-specific agonists are that they are poorly standardized and difficult to produce, leading to large batch-to-batch variability. Their application in a diagnostic setting would require extensive cross-calibration. As fibrillar collagen suspensions are unsuitable for flow cytometry assays, the critical need for the development of a novel, stable GPVI agonist is underscored most for flow cytometry.

Specific heavy-chain variable domain antibodies, or nanobodies, are small heavy-chain only antibodies derived from camelids such as llamas. Nanobodies retain the antigen specificity of the parental antibody, are highly stable, and can be efficiently produced on a large scale. This makes nanobodies ideal candidates for diagnostic purposes [15]. Within the SYMPHONY consortium [16], one of the aims is the improvement of laboratory diagnostics for patients with inherited bleeding disorders. In the current study, we developed a nanobody that specifically targets the extracellular domain of platelet GPVI. Based on the tetramer structure of convulxin, we produced a tetramer GPVI nanobody that induces platelet activation. We here describe the developmental and functional characterization and clinical validation of this nanobody-based GPVI-specific platelet agonist.

## 2 Methods

### 2.1 Subjects

#### 2.1.1 Healthy donors

Healthy controls were recruited among personnel and students at University Medical Center Utrecht by the MiniDonor biobank facility of the University Medical Center Utrecht (Biobank number 18-774) and were free of nonsteroid anti-inflammatory drugs. Approval was obtained from the local ethics review board, and all participants provided written informed consent.

#### 2.1.2 Patients with suspected primary hemostasis disorder

People with a (suspected) platelet disorder in the Netherlands were included in the Thrombocytopathy in the Netherlands study (study number NL53207.041.15), a nationwide cross-sectional study on disease phenotyping, diagnostics, and genetics at the University Medical Center Utrecht [17,18]. Exclusion criteria were von Willebrand disease and a coagulation factor deficiency. Patients came for a single hospital visit, and blood was collected for whole blood analysis, aggregation with 4 agonists, platelet surface receptor expression, nucleotide content, and genetic analysis. All measurements were done in the same sample, nonblinded. Medical ethical committee approval was obtained, and all patients provided written informed consent.

#### 2.1.3 Blood samples

In all participants, blood was collected from the antecubital vein in 3.2% trisodium citrate vacutainer tubes (BD) through phlebotomy. Blood was processed within 1 to 4 hours after collection. Platelet-rich plasma (PRP) was obtained by centrifugation at 160 × *g* without brake at room temperature (RT) for 15 minutes. Platelet-poor plasma (PPP) was obtained by centrifugation of the remainder of the blood at 2000 × *g* for 10 minutes at RT. Washed platelets were isolated from PRP, as described previously [19]. PRP was acidified and centrifuged at 340 × *g* for 15 minutes. The platelet pellet was resuspended in 4-(2-hydroxyethyl)-1-piperazineethanesulfonic acid (HEPES)-Tyrode’s buffer, pH 6.5, with 10 ng/mL prostacyclin. Platelets were centrifuged again at 340 × *g* for 15 minutes and resuspended in HEPES-Tyrode’s buffer, pH 7.3 to a platelet count of 200 × 10^9^/L. After isolation, washed platelets were allowed to rest for 30 minutes at RT.

### 2.2 Nanobody selection

A detailed description of nanobody selection can be found in the Supplementary Material. Lead nanobody D2 (NbD2) had the highest apparent affinity and was used for the current study.

### 2.3 Nanobody production and purification

The DNA sequence of anti-GPVI clone D2 was designed as a monomer or as a dimer with a 5-residue glycine-serine linker (GGGGS) codon optimized for production in *Escherichia coli* and ordered as a GeneBlock (Integrated DNA Technologies) with 5ʹ BamHI and 3ʹ NotI restriction sites. NbD2 dimer was then cloned into the pTH4.click production vector, which encodes a C-terminal Myc-tag for detection, a flexible glycine-serine linker, the LPETG sortase recognition sequence, and a His-tag for purification [20]. Nanobodies were expressed in BL21 Star (DE3)pLysS One Shot Chemically Competent *E. coli* (Invitrogen) in a bioreactor with autoinduction medium (1% tryptone, 0.5% yeast extract, 25 mM [NH_4_]_2_SO_4_, 50 mM KH_2_PO_4_, 50 mM Na_2_HPO_4_, 54 mM glycerol, 2.8 mM glucose, 5.6 mM lactose, 1 mM MgSO_4_) with 100 μg/mL ampicillin and 34 μg/mL chloramphenicol at RT overnight. Bacteria were harvested from the bioreactor and centrifuged at 5000 × *g* for 15 minutes at 4 °C. Pelleted bacteria were resuspended in 25 mM HEPES, 500 mM NaCl, pH 7.8 with 1 μg/mL DNAse, and 10 μM MgCl_2_. Bacteria were lysed with 3 subsequent freeze-thaw cycles in liquid nitrogen at 37 °C, followed by incubation with lysozyme from chicken egg white (Sigma-Aldrich) for 10 minutes at 37 °C. Lysed bacteria were centrifuged for 1 hour at 13,000 × *g* at 4 °C to pellet insoluble material, and nanobodies were purified from the supernatant with immobilized metal affinity chromatography on TALON Sepharose (Cytiva). All nanobodies were separated from remaining impurities with size-exclusion chromatography (SEC) on a HiLoad 26/600 75 pg gel column (Cytiva).

### 2.4 Tetramer generation with copper-free click chemistry

NbD2 dimers were labeled with a C-terminal azide using a sortagging procedure, as described [20]. In short, 100 μM purified NbD2 dimer was incubated with 1 μM sortase A (SrtA) and 1 mM H-(Gly)_3_-Lys(N3)-OH∗HCl (IRIS Biotech GmbH) for 2 hours at 25 °C to replace the C-terminal His-tag with a C-terminal azido group. Unlabeled dimers and SrtA were removed with immobilized metal affinity chromatography on TALON Sepharose. Next, azide-conjugated dimers were incubated for 18 hours at RT with the bivalent DBCO-PEG4-DBCO linker (BroadPharm) to generate tetramers. Tetramers were separated from unclicked dimers with SEC on a HiLoad 26/600 200 pg gel column. Purity was assessed with sodium dodecyl sulfate–polyacrylamide gel electrophoresis and Coomassie blue staining.

### 2.5 Determination of binding affinity to recombinant GPVI

The binding of NbD2 to soluble GPVI (sGPVI) was analyzed with surface plasmon resonance on a Biacore T100 system (Cytiva). A monoclonal anti–c-Myc antibody (clone 9E10; in-house produced; 50 μg/mL) was immobilized on reference and measurement flow channels of a CM5 sensor chip (Cytiva) with amine-coupling chemistry, according to the manufacturer’s instructions. Binding experiments were performed in flow buffer (10 mM HEPES, 150 mM NaCl, pH 7.4 with 0.05% Tween 20). NbD2 dimers (12.5 μg/mL) were captured on the measurement flow channels at a flow rate of 30 μL/min, followed by injection of sGPVI (0, 5, 10, 25, 50, and 100 nM) into reference and measurement channels at a flow rate of 30 μL/min for 1 minute to allow analysis of 1:1 protein interactions without contributions of avidity. Dissociation was monitored for 10 minutes. All flow channels were regenerated with 100 mM glycine, pH 2.7, at a flow rate of 30 μL/min for 1 minute, followed by a 10-minute stabilization period. Sensorgrams were adjusted for signal in the reference channel, and binding kinetics were determined by fitting the data to a 1:1 Langmuir binding model with Biacore T100 Evaluation software, version 2.0.4 (Cytiva).

### 2.6 Light transmission aggregometry

The aggregation of platelets was assessed with light transmission aggregometry (LTA) in a Chrono-Log model 700 (Kordia). The platelet count in PRP was adjusted to 200 × 10^9^/L with PPP. PPP was used as a blank in the aggregometer. To assess inhibition of aggregation by NbD2 monomers and NbD2 dimers, PRP was stimulated with either an excess (1 μM) NbD2 dimers alone or PRP was preincubated for 15 minutes at 37 °C at 900 rpm with 400 nM NbD2 monomers followed by 5 μg/mL Collagen Reagens HORM suspension (Takeda production site in Linz) or 1 μM D2 dimers followed by either 4 μg/mL HORM collagen or 1 μg/mL CRP-XL (CambCol Laboratories). Washed platelets (200 × 10^9^/L) were stimulated with 50 μg/mL fibrin directly or after preincubation with either 1 μM NbD2 dimers alone or in the presence of 300 μM Gly-Pro-Arg-Pro acetate (Sigma-Aldrich) to inhibit fibrin polymerization for 15 minutes at 37 °C at 900 rpm. Fibrin was prepared as described previously [21]. In short, 1 mg/mL fibrinogen in 0.06 M potassium phosphate buffer pH 6.8 was incubated with 2.5 U/mL human α thrombin for 1 hour at RT. The clot was washed in the same buffer, and 50 μM Phe-Pro-Arg-chloromethylketone (Prolytix) was added to inactivate thrombin. Fibrin polymers were dissolved in 0.02 M acetic acid. To investigate whether fibrin activates platelets through GPVI and initiates fibrinogen-dependent platelet aggregation through α_IIb_β_3_ rather than fibrin-dependent and α_IIb_β_3_-independent agglutination, 500 μM D-Arg-Gly-Asp-Trp (dRGDW; Bachem) was added to platelets to inhibit α_IIb_β_3_, followed by stimulation with fibrin.

To study the ability of NbD2 tetramers to initiate platelet aggregation, PRP was stimulated with 18 nM NbD2 tetramers or 1 μg/mL CRP-XL. To assess whether NbD2 tetramers are useful in diagnostics, platelet responses toward 1, 2, and 18 nM NbD2 tetramers were obtained in inherited platelet function disorder (IPFD) patients and compared with responses toward 1 and 4 μg/mL HORM collagen. Reference ranges for these agonists were determined in healthy donors.

### 2.7 Flow cytometry assays

Platelet surface expression of GPVI and platelet reactivity toward GPVI agonists were assessed with flow cytometry as described [22]. In short, whole blood was incubated with fluorophore-conjugated nanobodies or antibodies, with or without platelet agonists, and incubated in the dark at 37 °C for 10 minutes. In some experiments, whole blood from healthy controls was incubated with 5 μM P2Y_12_ platelet inhibitor cangrelor tetrasodium salt (Sigma-Aldrich) or 200 μM platelet cyclo-oxygenase 1 inhibitor indomethacin (Sigma-Aldrich) at RT for 30 minutes prior to use. Reactions were stopped by fixation with fixative buffer (137 mM NaCl, 2.7 mM KCl, 1.12 mM NaH_2_PO_4_, 1.15 mM KH_2_PO_4_, 10.2 mM Na_2_HPO_4_, 4 mM EDTA, 1.11% formaldehyde, pH 6.8) at RT for 20 minutes in the dark. After fixation, samples were diluted 1:1 (v/v) in HEPES-buffered saline (HBS: 10 mM HEPES, 150 mM NaCl, 1 mM MgSO_4_, 5 mM KCl, pH 7.4) and analyzed on a BD FACSCanto II Flow Cytometer (BD Biosciences). Platelets were gated based on forward and sideward scatter, as well as CD42b (GPIbα) expression. Platelet P-selectin expression and fibrinogen binding were assessed as described [22] to determine platelet reactivity. Platelet agonists were NbD2 dimers (0-30 nM), NbD2 tetramers (0-30 nM), ADP (50 μM), or CRP-XL (1 μg/mL) as indicated. For analysis of the inhibition of platelet reactivity toward CRP-XL with NbD2 dimers, whole blood was added to HBS with D2 dimers (0-30 nM), incubated at 37 °C for 10 minutes and stimulated with CRP-XL (1 μg/mL) at 37 °C for another 10 minutes. For detection of platelet GPVI with NbD2 dimers, whole blood was added to HBS with AlexaFluor 647-conjugated NbD2 (0-74.2 nM) and Rhodophyta-phycoerythrin (R-PE)-conjugated anti-GPIbα nanobody (clone 17; in-house produced; 15 μg/mL). In whole blood from IPFD patients, P-selectin expression and fibrinogen binding were assessed after stimulation with 18 nM NbD2 tetramer or 1 μg/mL CRP-XL. Data are expressed as median fluorescent intensity (MFI).

### 2.8 Inhibition assay

Competition between NbD2 monomers and CRP-XL on sGPVI was assessed using enzyme-linked immunosorbent assay. Soluble GPVI (2 μg/mL) in coating buffer (15 mM Na_2_CO_3_, 35 mM NaHCO_3_, 3 mM NaN_3_) was coated on a MaxiSorp 96-well plate (Nunc) for 30 minutes at RT under gentle agitation and then overnight at 4 °C. Wells were emptied and blocked for 1 hour with a blocking buffer (2% phosphate buffer saline–bovine serum albumin+ 0.05% Tween 20). All incubation steps were performed at RT and under gentle agitation. A nonsaturating concentration of NbD2 monomers (2.5 nM) together with a concentration series of CRP-XL (20-0 μg/mL) in blocking buffer were added to the wells and incubated for 1 hour. After washing, NbD2 was detected with monoclonal mouse anti–c-Myc IgG1 (clone 9E10; in-house produced; 1 μg/mL) for 1 hour. After washing, wells were incubated with polyclonal horseradish peroxidase-conjugated rabbit anti-mouse IgG (DAKO; 1:1000) for 1 hour. Wells were washed and stained with 50 μL 3,3ʹ,5,5ʹ-tetramethylbenzidine. Substrate conversion was stopped by adding 25 μL 0.3 M sulfuric acid, and absorbance was measured at 450 nm in a SpectraMax iD3 (Molecular Devices).

### 2.9 Measurement of protein phosphorylation

Measurement of protein phosphorylation was performed as described in Martin et al. [23]. Washed platelets were diluted to 400 × 10^9^/L and preincubated with 500 μM dRGDW to prevent platelet aggregation and with or without 10 μM Src inhibitor dasatinib (AbMole BioScience) or 1 μM SYK inhibitor PRT-060318 (Selleck Chemicals) for 30 minutes at RT. Platelets were stimulated with either 1 μg/mL CRP-XL, 18 nM NbD2 dimer, or 18 nM NbD2 tetramer, remained unstimulated, or were treated with dimethyl sulfoxide. After, platelets were lysed in reducing 3× sample buffer (15.5% glycerol, 96.8 mM Tris-HCl, pH 6.8, 0.6% sodium dodecyl sulfate, 0.003% bromophenol blue, 25 mM dithiothreitol) and heated for 10 minutes at 95 °C. Samples were run on a precast Bolt 4% to 12% Bis-Tris Plus polyacrylamide gel (Invitrogen) in 3-morpholinopropane-1-sulphonic acid buffer at 100 V. Proteins were transferred to an Immobilon-FL 0.45 μm PVDF membrane (Millipore) at 125 V. Phosphorylation of signal transduction proteins was detected with monoclonal mouse anti-phosphotyrosine (clone PY20; Biosource; 1:500) and monoclonal rabbit anti-phosphoSYK Tyr 525/526 (clone F.724.5; Invitrogen; 1:500). Monoclonal mouse anti-SYK (clone 4D10; Santa Cruz; 1:200) and polyclonal rabbit anti-GAPDH (Abcam; 1:1000) were used as lane loading controls. Secondary antibodies were goat anti-mouse AlexaFluor 680 (Thermo Fisher Scientific) and goat anti-rabbit IRDye800 (LI-COR) (1:10,000). Western blots were scanned using the Odyssey M fluorescence imaging system (LI-COR).

## 3 Results

### 3.1 Anti-GPVI NbD2 blocks CRP-XL-induced platelet aggregation

**Figure 1:**
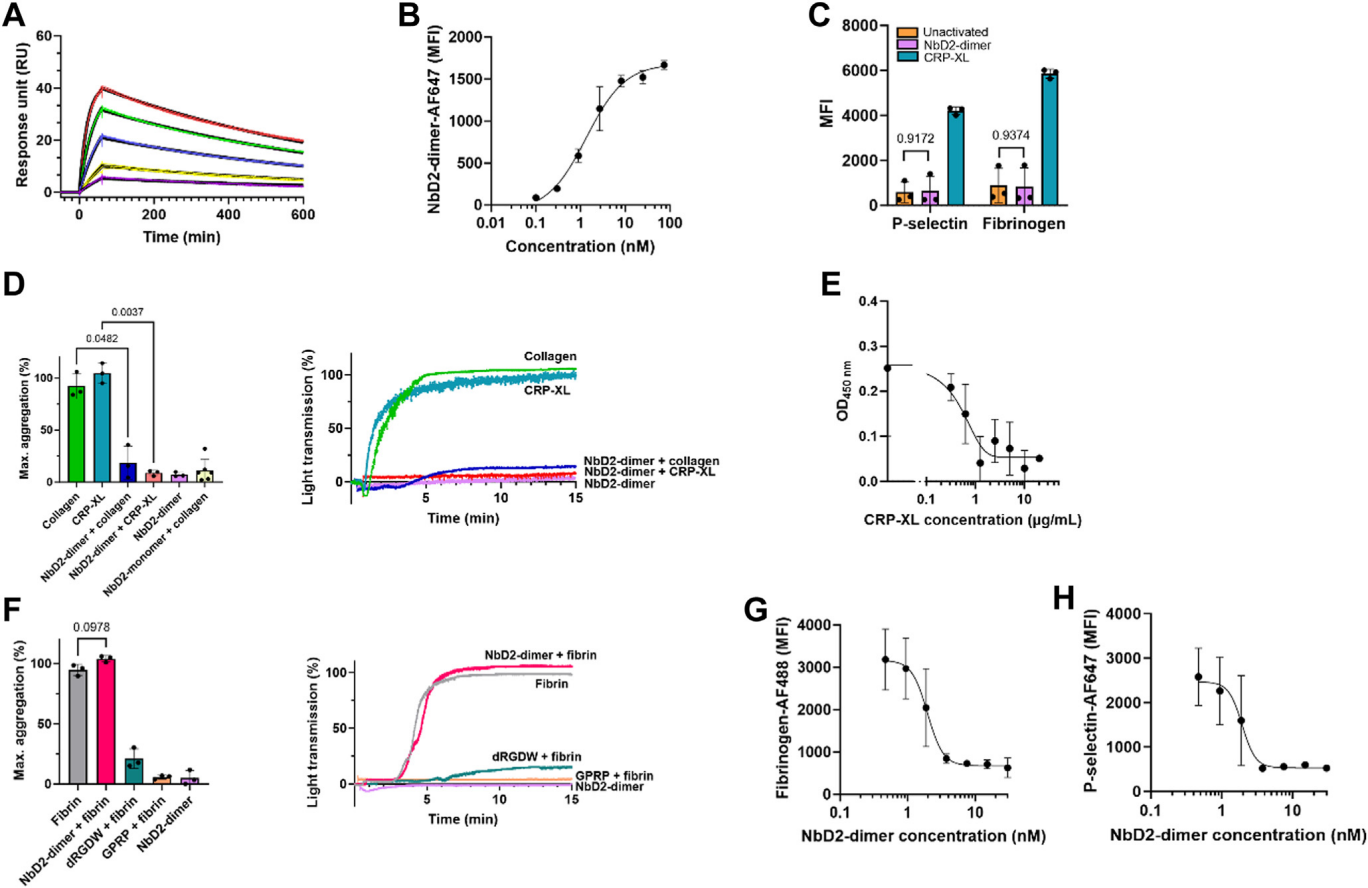
Antiglylcoprotein (GP)VI nanobody D2 (NbD2) blocks cross-linked collagen-related peptide (CRP-XL)-induced platelet aggregation. (A) Affinity of NbD2 dimers (5, 10, 25, 50, and 100 nM) to soluble GPVI was studied with surface plasmon resonance (*n* = 3). Colored lines indicate binding data. Solid black lines indicate fitted data. (B) Binding of NbD2 dimers was assessed in a flow cytometer. Whole blood from healthy donors (*n* = 3) was incubated for 10 minutes with AlexaFluor (AF)647-conjugated NbD2 dimer. Data are expressed as median fluorescent intensity (MFI). (C) Human whole blood from healthy donors was stimulated with either 1 μM NbD2 dimer, 1 μg/mL CRP-XL, or buffer for 10 minutes at 37 °C (*n* = 3). P-selectin expression and fibrinogen binding were assessed using flow cytometry. (D) Platelet-rich plasma from healthy donors was preincubated with either 1 μM NbD2 dimer alone or 1 μM NbD2 dimer, followed by stimulation with 4 μg/mL collagen or 1 μg/mL CRP-XL (*n* = 3) or 400 nM NbD2 monomer followed by 5 μg/mL collagen (*n* = 6) in a light transmission aggregometer. Aggregation was monitored for 15 minutes at 37 °C at 900 rpm. Bar graphs represent the maximum amplitude of aggregation. Representative traces of a single donor are shown. (E) sGPVI was incubated with 2.5 nM NbD2 in the presence of CRP-XL (0 to 20 μg/mL), and residual NbD2 binding was assessed (*n* = 3). Optical density (OD) was measured at 450 nm and plotted against CRP-XL concentration. (F) Human-washed platelets (*n* = 3) were preincubated with either 1 μM NbD2 dimer, 500 μM D-Arg-Gly-Asp-Trp (dRGDW), or 300 μM Gly-Pro-Arg-Pro acetate (GPRP), followed by stimulation with 50 μg/mL fibrin in a light transmission aggregometer. Aggregation was monitored for 15 minutes at 37 °C at 900 rpm. Bar graphs represent the maximum amplitude of aggregation. Representative traces of a single donor are shown. (G, H) Whole blood from healthy donors (*n* = 3) was preincubated with NbD2 dimers as indicated, followed by stimulation with 1 μg/mL CRP-XL. (G) Platelet P-selectin expression and (H) fibrinogen binding were assessed in a flow cytometer, and MFIs were plotted as a function of NbD2 dimer concentration. Statistical analyses were performed with a 1-way analysis of variance (anova) with Šidák correction. Error bars represent mean ± SD.

Nanobodies directed against GPVI were selected from phage display libraries obtained from 2 llamas immunized with the extracellular domain of human GPVI. NbD2 was identified as the best binder to immobilize GPVI and was used for further characterization. First, binding kinetics were analyzed with surface plasmon resonance analysis (Figure 1A). NbD2 showed a mean (SD) association rate of 5.1 (0.5) × 10^5^ M^-1^s^-1^, a mean (SD) dissociation rate of 1.3 (0.1) × 10^-3^ s^-1^, and a mean (SD) dissociation constant of 2.5 (0.4) nM, confirming that NbD2 has a high affinity for recombinant human GPVI. Next, the ability of D2 dimers to bind to native platelet GPVI was assessed with flow cytometry in human whole blood (Figure 1B). NbD2 dimers showed dose-dependent binding to platelet GPVI, confirming that NbD2 recognizes native human GPVI on platelets (dissociation constant 1.3 nM). Platelet activation via GPVI requires receptor clustering. To explore whether ligation of GPVI with NbD2 dimers induced platelet activation, human whole blood from healthy donors was incubated with NbD2 dimers, followed by analysis of P-selectin expression and fibrinogen binding with flow cytometry (Figure 1C). No increase in P-selectin expression or fibrinogen binding was observed after stimulation with NbD2 dimers, whereas stimulation with CRP-XL under the same conditions resulted in substantial P-selectin expression and fibrinogen binding. Similar results were obtained with LTA (Figure 1D). While stimulation with collagen or CRP-XL resulted in full platelet aggregation, platelet aggregation did not occur when PRP was stimulated with a saturating concentration (1 μM) of NbD2 dimers. As this could indicate that NbD2 recognizes an epitope remote from the collagen-binding site on GPVI, competition between collagen or CRP-XL and NbD2 dimers was assessed (Figure 1D). No aggregation occurred after stimulation with CRP-XL when platelets were preincubated with NbD2 dimers, and hardly any aggregation occurred after stimulated with collagen when platelets were preincubated with NbD2 monomers or NbD2 dimers, suggesting that NbD2 recognizes an epitope on GPVI overlapping with, or in close proximity to, the CRP-XL and collagen-binding site. To confirm that NbD2 binds to the collagen-binding site on GPVI, we performed a competitive enzyme-linked immunosorbent assay of NbD2 and CRP-XL on sGPVI. In line with our observations on the functional effects of NbD2, CRP-XL competed with NbD2 monomers for binding to sGPVI (Figure 1E), suggesting the collagen and NbD2 binding sites overlap.

As fibrin has also been described to activate platelets in a GPVI-dependent manner [24], we assessed the ability of NbD2 dimers to inhibit fibrin-induced platelet activation (Figure 1F). Stimulation of washed platelets with fibrin resulted in full aggregation, which was severely abrogated in the presence of fibrinogen receptor inhibitor dRGDW, confirming that stimulation of platelets with fibrin results in platelet activation and fibrinogen- and α_IIb_β_3_-dependent aggregation, rather than fibrin-dependent, α_IIb_β_3_-independent platelet agglutination [25]. Fibrin-dependent platelet aggregation required polymerized fibrin, as aggregation did not occur in the presence of fibrin polymerization inhibitor Gly-Pro-Arg-Pro acetate. In contrast with the effect of NbD2 dimers on collagen-induced platelet aggregation, preincubation with NbD2 dimers failed to inhibit fibrin-initiated platelet aggregation.

Having established that NbD2 specifically blocks the collagen-binding site, we further characterized the inhibitory effect of NbD2 dimers on GPVI-mediated platelet activation. Hereto, human whole blood was preincubated with NbD2 dimers and stimulated with CRP-XL, followed by analysis of P-selectin expression and fibrinogen binding with flow cytometry (Figure 1G, H). The addition of NbD2 dimers resulted in a dose-dependent reduction in P-selectin expression and fibrinogen binding after stimulation with CRP-XL, with a mean (95% CI) half-maximal inhibitory concentration of 1.9 (0.1-2.8) nM for P-selectin expression and a mean (95% CI) half-maximal inhibitory concentration of 2.0 (1.1-2.7) nM for fibrinogen binding.

### 3.2 Tetramers of anti-GPVI NbD2 induce platelet activation

**Figure 2:**
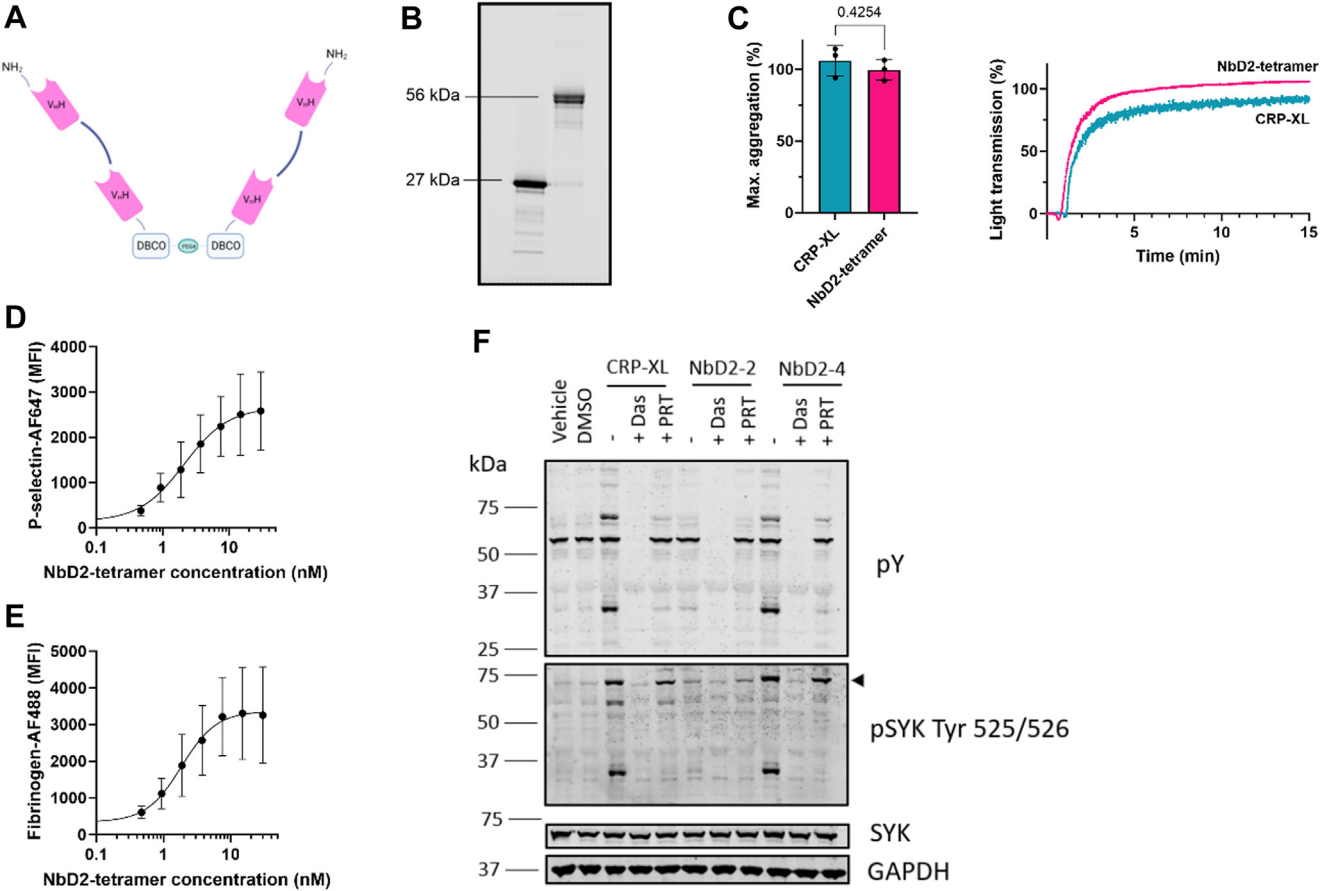
Tetramers of anti-glycoprotein VI nanobody D2 (NbD2) induce platelet aggregation. (A) A C-terminal azido group was introduced into NbD2 dimers with sortagging, followed by the formation of NbD2 tetramers with a DBCO-PEG4-DBCO (DBCO) linker with copper-free click chemistry. (B) Coomassie blue staining of NbD2 dimers (27 kDa) before sortase A treatment and NbD2 tetramers (56 kDa) after sortase A treatment, DBCO-linking and size-exclusion chromatography. (C) Platelet-rich plasma from healthy donors (*n* = 3) was stimulated with either 18 nM NbD2 tetramers or 1 μg/mL cross-linked collagen-related peptide (CRP-XL). Aggregation was monitored for 15 minutes at 37 °C. Bar graphs represent the maximum amplitude of aggregation. Representative traces of a single donor are shown. (D, E) Whole blood from healthy donors (*n* = 3) was preincubated with NbD2 tetramers as indicated. (D) Platelet P-selectin expression and (E) fibrinogen binding were assessed in a flow cytometer, and median fluorescent intensities (MFI) were plotted as a function of NbD2 tetramer concentration. (F) Signal transduction in platelet lysates was assessed using Western blot. Platelets were stimulated with 1 μg/mL CRP-XL, 18 nM NbD2 dimers (NbD2-2), or 18 nM NbD2 tetramers (NbD2-4) for 10 minutes at 37 °C with or without 10 μM Src inhibitor dasatinib (Das) or 1 μM SYK inhibitor PRT-060318 (PRT). phosphoSYK (pSYK) and phosphotyrosine (pY) were detected. The arrow indicates SYK. SYK and GAPDH were used as lane loading controls. Statistical analysis was performed with a Student’s *t*-test. Error bars represent mean ± SD. AF647/488, AlexaFluor 647/488; V_H_H, heavy-chain variable domain antibodies. DMSO, dimethyl sulfoxide.

Dimerization of GPVI was insufficient to induce platelet activation. To investigate whether higher-order multimers would induce platelet activation, we engineered NbD2 tetramers. Hereto, NbD2 dimers were treated with SrtA to introduce a glycinilated azide tail. C-termini of 2 NbD2 dimers were linked with copper-free click chemistry to ensure optimal availability of the complementarity-determining regions in NbD2. (Figure 2A). Purification of tetramers yielded a band of approximately 56 kDa, twice the size of dimers (27 kDa) on a Coomassie blue staining (Figure 2B).

To assess whether NbD2 tetramers were capable of inducing platelet activation, NbD2 tetramers were compared with CRP-XL using LTA. NbD2 tetramers showed immediate and full platelet aggregation, similar to CRP-XL, with a maximum aggregation of 99.5 ± 7.1% in 3 healthy volunteers with NbD2 tetramers compared with 106.0 ± 10.6% with CRP-XL (Figure 2C). The platelet-activating effect of NbD2 tetramers was further investigated in a whole blood flow cytometry assay. NbD2 tetramers induced a dose-dependent increase in P-selectin expression and fibrinogen binding with a mean (95% CI) half-maximal effective concentration of 2.1 (1.2-6.7) nM and 1.8 (1.0-3.8) nM, respectively (Figure 2D, E). Combined, these data indicate that NbD2 tetramers act as platelet agonists in LTA and flow cytometry.

In order to investigate why NbD2 dimers do not induce platelet activation, and tetramers do, signaling events were investigated after stimulation with CRP-XL, NbD2 dimers, or NbD2 tetramers with Western blotting (Figure 2F). NbD2 tetramers induced tyrosine phosphorylation events, as well as substantial SYK phosphorylation, similar to CRP-XL, whereas NbD2 dimers did not. Src inhibitor dasatinib and SYK inhibitor PRT-060318 inhibited tyrosine phosphorylation after stimulation with NbD2 tetramers and CRP-XL. These results demonstrate that NbD2 tetramers stimulate potent platelet activation through GPVI, similar to CRP-XL, while NbD2 dimers fail to induce intracellular signaling events.

### 3.3 NbD2 tetramers can be used as GPVI agonists during the diagnostic follow-up of patients with a suspected platelet function disorder

**Figure 3:**
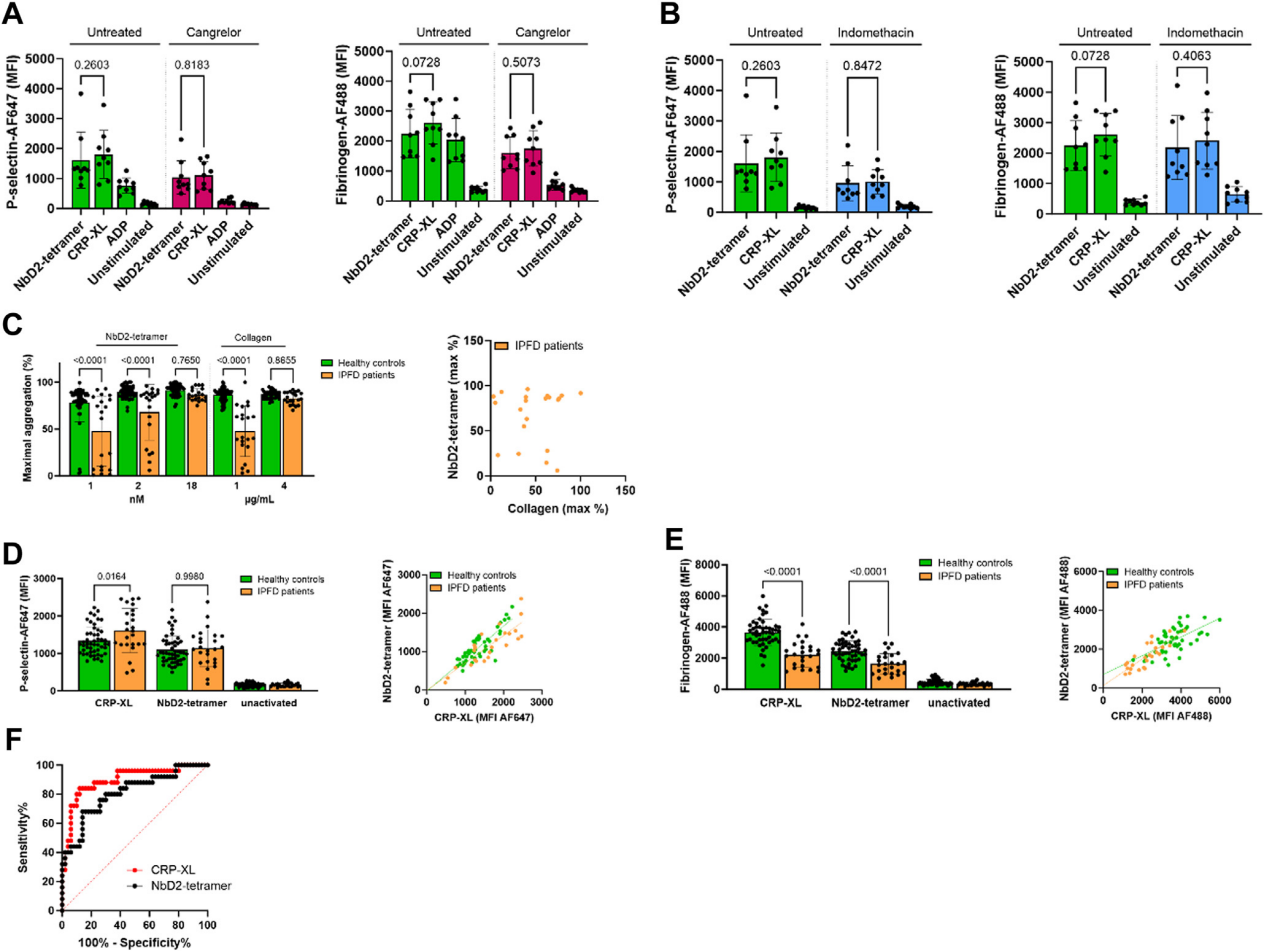
Nanobody D2 (NbD2) tetramer can be used as a glycoprotein VI agonist during the diagnostic follow-up of patients with a suspected platelet function disorder. (A, B) Human whole blood was incubated with 5 μM cangrelor or 200 μM indomethacin for 30 minutes at room temperature. Platelet activation was allowed for 10 minutes at 37 °C with either cross-linked collagen-related peptide (CRP-XL), NbD2 tetramers, or adenosine diphosphate (ADP), and P-selectin expression and fibrinogen binding was assessed with median fluorescent intensity (MFI) of fluorescently labeled nanobodies. Bar graphs represent mean P-selectin expression and fibrinogen binding in healthy donors (*n* = 9). (C) Platelet-rich plasma from patients with an inherited platelet function disorder (IPFD; *n* = 21) and healthy controls (*n* = 50) were stimulated with either 1, 2, or 18 nM NbD2 tetramer or 1 or 4 μg/mL collagen. Aggregation was monitored for 15 minutes at 37 °C. Bar graphs represent the maximum amplitude of aggregation. A correlation plot is shown for maximum aggregation with 2 nM NbD2 tetramer and 1 μg/mL collagen. (D, E) Whole blood from IPFD patients (*n* = 25) and healthy controls (*n* = 50) were stimulated with either 18 nM NbD2 tetramer or 1 μg/mL CRP-XL for 10 minutes at 37 °C. (D) P-selectin expression and (E) fibrinogen binding were assessed using flow cytometry. Data are expressed as MFI. Correlation plots for both agonists are shown for P-selectin expression and fibrinogen binding. (F) Area under the receiver operating characteristic for CRP-XL vs NbD2 tetramer in flow cytometric fibrinogen binding in healthy controls and IPFD patients. Statistical analyses were performed with 1-way anova with Šidák correction. Error bars represent mean ± SD. AF647/488, AlexaFluor 647/488.

Next, we investigated whether NbD2 tetramers can be used to detect platelet function disorders. Hereto, platelets were treated with P2Y_12_ inhibitor cangrelor or cyclo-oxygenase 1 inhibitor indomethacin to mimic platelet disorders, followed by stimulation with either CRP-XL or NbD2 tetramers. P-selectin expression and fibrinogen binding were assessed using flow cytometry. Both P-selectin and fibrinogen binding were decreased in cangrelor-treated blood, but P-selectin expression was decreased, while fibrinogen binding was normal in indomethacin-treated blood compared with untreated blood. No differences in P-selectin expression or fibrinogen binding after stimulation with CRP-XL or NbD2 tetramers were observed in both untreated and inhibited samples; in untreated samples (Figure 3A, B), the mean ± SD P-selectin expression was 1613 ± 934 with NbD2 tetramers compared with 1805 ± 801 for CRP-XL (*P* = .26). For fibrinogen binding, this was 2249 ± 812 and 2602 ± 698, respectively (*P* = .07). When platelets were inhibited with cangrelor (Figure 3A), P-selectin expression was 1036 ± 561 with D2 tetramers and 1103 ± 466 with CRP-XL (*P* = .82). For fibrinogen binding, D2 tetramers resulted in an MFI of 1606 ± 527 compared with 1754 ± 590 with CRP-XL (*P* = .51). Lastly, indomethacin-treated blood (Figure 3B) resulted in P-selectin expression of 961 ± 576 with NbD2 tetramers compared with 1010 ± 402 for CRP-XL (*P* = .85) and fibrinogen binding of 2177 ± 1050 and 2406 ± 935 (*P* = .41). The latter was not decreased compared with healthy controls for both agonists. These data suggest that NbD2 tetramers perform similarly to CRP-XL and might be suitable platelet agonists for diagnostic purposes.

To further evaluate the diagnostic performance of platelet function testing with NbD2 tetramers, LTA and flow cytometry assays were performed in blood from 25 patients with a confirmed IPFD and in 50 healthy controls (Table). Five IPFD patients were excluded from the LTA analysis due to thrombocytopenia. Many IPFD patients showed decreased aggregation (mean maximum aggregation, 48 ± 27%) after stimulation with a low concentration of collagen (1 μg/mL) compared with healthy controls (86 ± 7%; *P* < .0001; Figure 3C). Similar results were obtained with low concentrations of NbD2 tetramer (1 and 2 nM; 48 ± 37% and 68 ± 30% in IPFD patients and 78 ± 20% and 89 ± 6% in healthy controls; *P* < .0001). A high concentration of collagen (4 μg/mL) resulted in normal platelet aggregation in both IPFD patients (83 ± 6%) and healthy controls (87 ± 4%; *P* = .87). The same was seen for a high concentration of NbD2 tetramer (18 nM; 87 ± 7% in IPFD patients and 91 ± 6% in healthy controls; *P* = .77). However, the maximum aggregation response observed with 2 nM NbD2 tetramer correlated poorly with the response toward 1 μg/mL collagen (Pearson r = .03 in IPFD patients; Figure 3C).

**Table:** Baseline demographics and clinical characteristics of participants with inherited platelet function disorders.

Characteristics	Inherited platelet function disorder (*n* = 25)
Sex, *n* (%)	
Males	10 (40)
Females	15 (60)
Age (y)	
Median	39
Range	21-75
Platelet count (×10^9^/L)	
Median	237
Range	53-464
Thrombocytopenia (*n*)	
100-150 × 10^9^/L	3
50-100 × 10^9^/L	3
MPV (fL)	
Median	8.4
Range	6.8-10.7
IPFD (*n*)	25
SPD^a^	18
Hermanksy–Pudlak	1
RUNX1 familial platelet disorder	2
Platelet-type bleeding disorder-17	1
XLTT	1
Familial thrombopathic thrombocytopenia	1
Unspecified platelet aggregation defect	2

IPFD, inherited platelet function disorder; LTA, light transmission aggregometry; MPV, mean platelet volume; SPD, storage pool disease; XLTT, X-linked thrombocytopenia with thalassemia.

a Platelet adenosine diphosphate content < 1.4 μmol/10^11^ platelets.

Platelet surface P-selectin expression after stimulation with CRP-XL was normal or increased in IPFD patients compared with healthy controls (Figure 3D), whereas fibrinogen binding was decreased in IPFD patients (2225 ± 799 for CRP-XL and 1647 ± 659 for NbD2 tetramers) compared with healthy controls (3665 ± 840 and 2463 ± 635; *P* < .0001; Figure 3E). P-selectin expression after CRP-XL stimulation correlated well with P-selectin expression after stimulation with NbD2 tetramers in healthy controls and IPFD patients (Pearson r = .85 in both healthy controls and IPFD patients), as did fibrinogen binding (Pearson r = .64 and .83, respectively; Figure 3D, E). The diagnostic performance of fibrinogen binding after stimulation with either agonist was similar, with an area under the receiver operating characteristic of 0.90 ± 0.04 for CRP-XL and 0.81 ± 0.05 for NbD2 tetramer (*P* = .21; Figure 3F). For both agonists, sensitivity was 40% (95% CI, 23.40%-59.26%), and specificity was 98% (95% CI, 89.50%-99.90%), with a positive likelihood ratio (LR) of 20.0 and negative LR of 0.61. Corresponding cutoff values were 2100 MFI for CRP-XL and 1311 MFI for NbD2 tetramer. This indicates that NbD2 tetramers can be used to discriminate patients with IPFD from healthy controls with a flow cytometric diagnostic assay based on fibrinogen binding to platelets, with a similar sensitivity and specificity as CRP-XL.

## 4 Discussion

This study shows that a tetrameric nanobody targeting platelet collagen receptor GPVI has the capability to activate platelets and can be utilized to study differences in platelet reactivity between IPFD patients and healthy controls. Based on our data, the nanobody recognizes an epitope that overlaps with the collagen-binding site on GPVI. While it effectively blocks GPVI-mediated activation by collagen when used as a dimer, it leads to full platelet activation in both LTA and flow cytometry-based platelet activation assays when used as a tetramer.

In previous studies using anti-GPVI nanobodies, it was shown that dimerization of GPVI is not sufficient for platelet activation, but tri- or tetramerization is [23,26]. Our data are in line with these observations, as our dimeric nanobody has an inhibiting effect on collagen or CRP-XL-induced platelet activation, and our tetrameric nanobody acts as an agonist.

Crystal structures of GPVI have shown that the primary binding site for collagen lies across the D1 domain β-sheet [27]. Our data suggest that the binding site of NbD2 on GPVI overlaps with the binding site on GPVI for collagen. Glenzocimab, an inhibitory antibody Fab-fragment against the D2 domain of GPVI that is currently under investigation in phase 2/3 clinical trials [28], was shown to prevent collagen binding to GPVI through steric hindrance [21]. Although NbD2 dimers (27 kDa) are smaller than glenzocimab (48 kDa), we cannot exclude steric hindrance, which plays a role in the effects of NbD2.

Fibrin is an additional ligand for GPVI, resulting in GPVI-mediated platelet activation [24]. Although the exact fibrin binding site on GPVI is not known, it is suggested that it is in close proximity to the collagen-binding site [29]. Our NbD2 dimers did not act as an inhibitor for fibrin-initiated platelet aggregation, suggesting that NbD2 binds remotely from the fibrin binding site. The relevance of fibrin binding to GPVI for diagnostics of platelet disorders remains to be determined.

Activation of platelets through GPVI is always included in diagnostic assays for platelet function disorders such as LTA and flow cytometry. The major ligand for GPVI, the matrix protein collagen, is insoluble and mostly used as poorly standardized suspensions of fibrillar collagen. Platelet disorders that show reduced platelet reactivity toward collagen include GPVI deficiency, either congenital or acquired due to autoantibodies [8,9], GPVI-related signaling defects [6], and defects in reinforcing pathways of platelet activation, such as SPDs [12]. Patients with GPVI deficiency show strongly reduced platelet aggregation with collagen and decreased P-selectin expression and fibrinogen binding in flow cytometry diagnostic workup. The effects of SPD on collagen-induced platelet aggregation are less pronounced, with normal LTA responses in many patients with SPD [30]. None of the patients in our study had GPVI deficiency, and 72% of the patients in our study had SPD. In line with reported LTA outcomes in SPD, many patients displayed normal LTA responses to collagen and NbD2 tetramers.

LTA outcomes for collagen and NbD2 tetramers did not correlate. A possible explanation for the discrepancy between NbD2 tetramers and collagen in LTA could be that collagen binds to both GPVI and α_2_β_1_, whereas NbD2 only interacts with GPVI. A deficiency in integrin α_2_β_1_ results in reduced collagen-mediated aggregation [31], indicating that the α_2_β_1_ signaling pathway also plays a role in collagen-induced platelet aggregation. As NbD2 dimers fully block collagen-induced platelet activation, any α_2_β_1_-mediated responses toward collagen are likely to occur secondary to GPVI-mediated signaling.

In sharp contrast with LTA data, platelet fibrinogen binding after stimulation with NbD2 tetramers correlated well with fibrinogen binding after stimulation with CRP-XL in flow cytometry. Platelet responses to the NbD2 tetramer were similar to those with the GPVI-specific agonist CRP-XL in flow cytometry in both IPFD patients and healthy controls. The diagnostic performance of both agonists was similar, with very good discrimination between healthy controls and IPFD patients, supporting the potential application of NbD2 tetramers in the evaluation of platelet function during the diagnostic workup of primary hemostasis defects.

Limitations of this pilot study on diagnostic performance are that our cohort of 25 IPFD patients was relatively small, analysis was not performed blind to diagnosis, and SPD was overrepresented, which may have introduced an overestimation of diagnostic performance. Sensitivity and specificity should, therefore, be interpreted with caution. However, the LR, a measure insensitive to prevalence, suggests NbD2 tetramers are suitable for diagnostic use. Our findings need to be confirmed in a larger prospective study in a relevant patient population. Platelet function analysis is performed to confirm or exclude platelet disorders in patients with clinical symptoms of a primary hemostasis defect characterized by a mucocutaneous bleeding tendency. Validation of NbD2 tetramers for diagnostic purposes will, therefore, require the evaluation of platelet function in a larger cohort of people with a mucocutaneous bleeding tendency. Such analyses are hampered by the lack of gold-standard tests for platelet function abnormalities.

Lastly, due to the stable and easily producible nature of nanobodies, this technique could be further developed for other receptors, as was already done by Martin et al. [23], who developed trivalent nanobody-based ligands for GPVI, C-type lectin-like receptor 2, and platelet endothelial aggregation receptor 1 and a tetravalent ligand for FcγRIIA. However, the diagnostic value of other platelet nanobody-based ligands would need to be determined.

In conclusion, we here show the development and functional characterization of a novel tetrameric GPVI-specific nanobody that may serve as a stable platelet agonist in diagnostic assays.

## Appendix

**TiN study group:** Erik Beckers, Maastricht, the Netherlands, Michiel Coppens, Amsterdam, the Netherlands, Jeroen Eikenboom, Leiden, the Netherlands, Louise Hooimeijer, Groningen, the Netherlands, Gerard Jansen, Rotterdam, the Netherlands, Roger Schutgens, Utrecht, the Netherlands, Rolf Urbanus, Utrecht, the Netherlands, and Minka Zivkovic, Utrecht, the Netherlands.

**SYMPHONY consortium:** Emile van den Akker, Amsterdam, the Netherlands, Wala Al Arashi, Rotterdam, the Netherlands, Ryanne Arisz, Rotterdam, the Netherlands, Lieke Baas, Utrecht, the Netherlands, Ruben Bierings, Rotterdam, the Netherlands, Maartje van den Biggelaar, Amsterdam, the Netherlands, Johan Boender, Amsterdam, the Netherlands, Anske van der Bom, Leiden, the Netherlands, Mettine Bos, Leiden, the Netherlands, Martijn Brands, Amsterdam, the Netherlands, Annelien Bredenoord, Utrecht, the Netherlands, Laura Bukkems, Amsterdam, the Netherlands, Lex Burdorf, Rotterdam, the Netherlands, Jessica Del Castillo Alferez, Amsterdam, the Netherlands, Michael Cloesmeijer, Amsterdam, the Netherlands, Marjon Cnossen, Rotterdam, the Netherlands, Mariëtte Driessens, Utrecht, the Netherlands, Jeroen Eikenboom, Leiden, the Netherlands, Karin Fijnvandraat, Amsterdam, the Netherlands, Kathelijn Fischer, Utrecht, the Netherlands, Geertje Goedhart, Leiden, the Netherlands, Tine Goedhart, Rotterdam, the Netherlands, Samantha Gouw, Amsterdam, the Netherlands, Rieke van der Graaf, Utrecht, the Netherlands, Masja de Haas, Amsterdam, the Netherlands, Lotte Haverman, Amsterdam, the Netherlands, Jan Hazelzet, Rotterdam, the Netherlands, Shannon van Hoorn, Rotterdam, the Netherlands, Elise Huisman, Rotterdam, the Netherlands, Nathalie Jansen, Utrecht, the Netherlands, Alexander Janssen, Amsterdam, the Netherlands, Sean de Jong, Hoofddorp, the Netherlands, Sjoerd Koopman, Amsterdam, the Netherlands, Marieke Kruip, Rotterdam, the Netherlands, Sebastiaan Laan, Leiden, the Netherlands, Frank Leebeek, Rotterdam, the Netherlands, Nikki van Leeuwen, Rotterdam, the Netherlands, Hester Lingsma, Rotterdam, the Netherlands, Moniek de Maat, Rotterdam, the Netherlands, Ron Mathôt, Amsterdam, the Netherlands, Felix van der Meer, Leiden, the Netherlands, Karina Meijer, Groningen, the Netherlands, Sander Meijer, Amsterdam, the Netherlands, Stephan Meijer, Den Haag, the Netherlands, Iris van Moort, Rotterdam, the Netherlands, Caroline Mussert, Rotterdam, the Netherlands, Hans Kristian Ploos van Amstel, Utrecht, the Netherlands, Suzanne Polinder, Rotterdam, the Netherlands, Diaz Prameyllawati, Rotterdam, the Netherlands, Simone Reitsma, Rotterdam, the Netherlands, Eliza Roest, Rotterdam, the Netherlands, Lorenzo Romano, Rotterdam, the Netherlands, Saskia Schols, Nijmegen, the Netherlands, Roger Schutgens, Utrecht, the Netherlands, Rolf Urbanus, Utrecht, the Netherlands, Carin Uyl, Rotterdam, the Netherlands, Jan Voorberg, Amsterdam, the Netherlands, Huan Zhang, Amsterdam, the Netherlands, and Minka Zivkovic, Utrecht, the Netherlands.
