# Supplementary material for: Functional characterization of a nanobody-based glycoprotein VI-specific platelet agonist
Source: Res Pract Thromb Haemost. 2024 Oct 3;8(7):102582. doi: 10.1016/j.rpth.2024.102582 (PMC11541698; doi:10.1016/j.rpth.2024.102582)
Supplement: Supplementary material [file mmc1.docx]

**Supplementary Material**

**Nanobody selection**

The recombinant soluble extracellular domain of human GPVI (sGPVI; amino acids 21-267) was produced as monomer and purified by U-protein express (Utrecht, the Netherlands). Two llamas (*Lama glama*) received 4 rounds of subcutaneous immunizations with 50 µg sGPVI at day 0, 14, 28 and 56. Blood was drawn at 3 months after immunization for peripheral blood B-lymphocyte isolation. Phage display libraries containing the sequences of all V_H_H regions of heavy-chain only antibodies were constructed by QVQ (Utrecht, the Netherlands), as described[^1^](https://sciwheel.com/work/citation?ids=13721598&pre=&suf=&sa=0&dbf=0). Nanobodies were selected as described[^2^](https://sciwheel.com/work/citation?ids=9964106&pre=&suf=&sa=0&dbf=0), with a few modifications. In short, Nunc PolySorp microtiter plates were coated with NeutrAvidin® (Life Technologies, Carlsbad, CA; 5 µg/mL) in 50 mM Na_2_CO_3_, 50 mM NaHCO_3_, 0.05% N_3_, pH 9.6 at 4°C overnight, washed and blocked with 1% bovine serum albumin (BSA) in 137 mM NaCl, 2.7 mM KCl, 9.2 mM Na_2_HPO_4_, 1.76 mM KH_2_PO_4_, pH 7.4 (PBS-BSA). All subsequent incubations took place at RT for 1 hour under gentle agitation on a plate shaker, unless otherwise indicated. GPVI was biotinylated with EZ-Link Sulfo-NHS-LC-Biotin according to manufacturer’s instructions (Life Technologies) and incubated in NeutrAvidin coated wells (1.5 µg/mL). Plates were washed and incubated with phages in PBS-BSA under gentle agitation for 2 hours. Plates were washed 15 times with PBS containing 0.1% Tween-20, followed by 3 times with PBS only. Bound phages were eluted with 0.1 M triethylamine, collected and transferred to neutralizing buffer (1 M Tris/HCl, pH 7.5, sterile). *Escherichia*  *coli* (*E. coli*) (strain TG1) were incubated with phages, plated on Yeast Tryptone Broth agar plates containing 2% glucose and 100 µg/mL ampicillin and grown overnight at 37ºC. Single colonies were picked, grown for 5 hours at 37ºC, followed by induction of nanobody production with isopropyl β-D-1-thiogalactopyranoside (IPTG, 1 mM) overnight. Binding of nanobodies to GPVI was assessed on microtiter plates with NeutrAvidin captured GPVI-biotin. GPVI-specific nanobodies were sequenced. Unique clones were produced in large quantity and purified with immobilized metal affinity chromatography (IMAC) on TALON® Superflow™ Sepharose. Lead nanobody D2 (NbD2) had the highest apparent affinity and was used for the current study.

**References**

[1.    de Maat S, van Dooremalen S, de Groot PG, Maas C. A nanobody-based method for tracking factor XII activation in plasma. *Thromb Haemost*. 2013;110(3):458-468. doi:10.1160/TH12-11-0792](https://sciwheel.com/work/bibliography/13721598)

[2.    van Moorsel MVA, Urbanus RT, Verhoef S, et al. A head-to-head comparison of conjugation methods for VHHs: Random maleimide-thiol coupling versus controlled click chemistry. *International Journal of Pharmaceutics: X*. 2019;1:100020. doi:10.1016/j.ijpx.2019.100020](https://sciwheel.com/work/bibliography/9964106)
